# Supplementary figures and images for: The Clinical Outcome of Early Periprosthetic Joint Infections Caused by Staphylococcus epidermidis and Managed by Surgical Debridement in an Era of Increasing Resistance
Source: Antibiotics (Basel). 2022 Dec 27;12(1):40. doi: 10.3390/antibiotics12010040 (PMC9854449; doi:10.3390/antibiotics12010040)

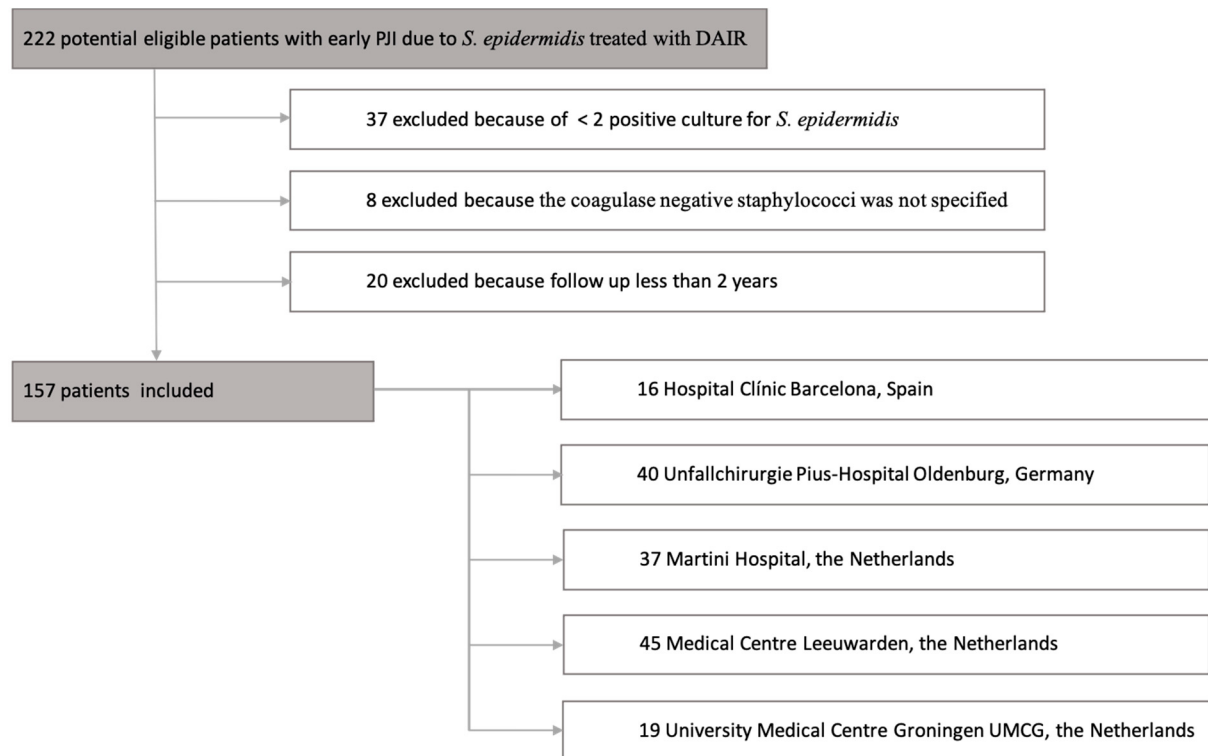

Figure S1. Flow chart patient inclusion.

Supplement: Supplementary file 1 [file antibiotics-12-00040-s001.zip › antibiotics-2048055-supplementary.pdf]
